# A positive feedback loop of ARF6 activates ERK1/2 signaling pathway via *DUSP6* silencing to promote pancreatic cancer progression: ARF6 regulates ERK1/2 pathway by inhibiting DUSP6

**DOI:** 10.3724/abbs.2022111

**Published:** 2022-08-19

**Authors:** Bingkai Xiao, Yue Zhang, Zekun Lu, Weibo Chen, Yong An, Guangchen Zu, Xiaowu Xu, Di Wu, Hao Yang, Yi Qin, Xuemin Chen

**Affiliations:** 1 Department of Hepatopancreatobiliary Surgery the Third Affiliated Hospital of Soochow University Changzhou 213000 China; 2 Department of Pancreatic Surgery Fudan University Shanghai Cancer Center Shanghai 200032 China

**Keywords:** ERK1/2 pathway, ARF6, DUSP6, pancreatic adenocarcinoma

## Abstract

ERK1/2 are essential proteins mediating mitogen-activated protein kinase signaling downstream of RAS in pancreatic adenocarcinoma (PDAC). Our previous study reveals that ARF6 plays a positive regulatory role in ERK1/2 pathway in a feedback loop manner. A significant part of the literature on ARF6 has emphasized its oncogenic effect as an essential downstream molecule of ERK1/2, and no research has been done on the regulation mechanisms of the feedback loop between ARF6 and the ERK1/2 signaling pathway. In the present study, we explore the gene network downstream of *ARF6* and find that DUSP6 may be the critical signal molecule in the positive feedback loop between ARF6 and ERK1/2. Specifically, to elucidate the negative correlations between ARF6 and DUSP6 in pancreatic cancer, we examine their expressions in pancreatic cancer tissues by immunohistochemical staining. Then the impact of DUSP6 on the proliferation and apoptosis of PDAC cells are investigated by gain-of-function and loss-of-function approaches. Mechanism explorations uncover that ARF6 suppresses the expression of DUSP6, which is responsible for the dephosphorylation of ERK1/2. Altogether, these results indicate that DUSP6 plays a tumor-suppressive role and acts as an intermediate molecule between ARF6 and ERK1/2 in PDAC cells, thereby forming a positive feedback loop.

## Introduction

Pancreatic ductal adenocarcinoma (PDAC) is a highly aggressive malignancy with poor survival, and the overall ratio of mortality to incidence is close to 1. Despite significant advances in cancer diagnosis and treatment during the past years, the overall 5-year survival rate for PDAC remains the lowest among all malignancies at 9% [1]. Due to the difficulty in early diagnosis, uncomplicated early metastasis, rapid development and lack of effective therapy, only 15%–20% of patients are eligible for surgical resection which is the only potentially curative option at present [ 2– 4] . Remarkably, several researchers have found that approximately 95% of pancreatic cancer patients harbor somatic mutations of the *KRAS* gene, which is considered the Everest of cancer research [ 5, 6] . *KRAS* mutations are known to activate the RAF-MEK-ERK pathway, which is regarded as a significant driving force in malignant transformation and tumor development and is closely associated with prognosis [ 7, 8] . Meanwhile, we found that *KRAS* silencing inhibited the proliferation and metastasis capacity of pancreatic cancer cells *in vitro* [9]. However, all the attempts to directly target *KRAS* or its downstream effector molecules like RAF1 have been shown unsuccessful or remain at a preliminary stage which shows that there is still no effective KRAS-targeted therapy available in the clinic [10]. Thus, clarifying the precise molecular mechanism of KRAS in the development of pancreatic cancer is a pivotal way to conquer it, and the inhibition of KRAS downstream targets is considered an effective strategy [11]. Research in this area is still evolving. For exemple, the perineural invasion in pancreatic ductal adenocarcinoma can be predicted by SVM classifier based on these 26 key features with a high accuracy of 0.94 evaluated with leave-one-out cross validation [12].

In a previous study, ARF6 was found to be a member of the Ras superfamily, which is induced by the KRAS-ERK1/2 pathway and promotes pancreatic cancer development by satisfying the increased energy needs and biosynthetic requirements of uncontrolled cell growth [13]. Unexpectedly, we found that silencing of *ARF6* inhibited the activation of ERK1/2 [14]. These findings indicated that ARF6 might play a dual role in the ERK pathway by acting as both a downstream signaling effector and a regulator of ERK activation. To sum up, there is a positive feedback loop between ARF6 and ERK1/2, leading to the promotion of proliferation and tumorigenesis in pancreatic cancer by amplifying the oncogenic effect of KRAS.

The DUSP family members were identified as classic tumor suppressors in various cancers [15]. DUSP proteins have been grouped in class I of the cysteine-based protein tyrosine phosphatase (PTP) family that carries a single catalytic PTP domain, which accounts for their ability to dephosphorylate both Ser/Thr- and Tyr-phosphorylated residues in proteins [ 16, 17] . And the capacity is the crucial factor for the inactivation of mitogen-activated protein kinases (MAPKs) [18]. Concisely, the 10 MAPK phosphatases can be clustered into three major subgroups. The first group is inducible nuclear mitogen-activated kinase phosphatases (MKPs), comprising DUSP1/MKP-1, DUSP2, DUSP4/MKP-2 and DUSP5. The second group includes DUSP6/MKP-3, DUSP7/MKP-X and DUSP9/MKP-4, all of which are cytoplasmic ERK-specific MKPs. The third group has three MKPs, including DUSP8, DUSP10/MKP-5 and DUSP16/MKP-7 that can inactivate p38 and JNK MAPKs [19]. DUSPs exert their tumor suppressor function in multiple aspects and multiple tumor types, including limiting the progression of the tumor, promoting tumor apoptosis, *etc*. Overexpression of DUSP8 was found to remarkably suppress the proliferation and migration of colorectal carcinoma cells (CRC) *in vitro* [20]; DUSP6/MKP-3 expression levels showed a negative correlation with the KRAS expression levels in non-small cell lung cancer (NSCLC) [21]. Meanwhile, the encoding gene, *DUSP*6, is located on 12q21-q22, the region commonly deleted hemizygously in pancreatic cancer [22]. Taken together, DUSPs exert their tumor suppressor function through complex interaction networks and some of them may be correlated with ARF6 expression. Therefore, more attention should he paid on the expressions of DUSPs in pancreatic cancer.

In the present study, we describe a positive feedback loop about ERK1/2-ARF6 pathway that can inhibit the expression of DUSP6 in pancreatic cancer cells, which is equivalent to removeing the inhibition of ERK1/2 pathway. Since some small molecules that specifically target ERK1/2-ARF6-DUSP6 axis have exhibited potential clinical application value, our findings might provide new strategies for the comprehensive treatment of pancreatic cancer.

## Materials and Methods

### Cell culture

The human pancreatic cancer cell lines PANC-1 and SW1990 were obtained from ATCC (Manassas, USA) and cultured according to standard ATCC protocols. 293T cells were purchased from Invitrogen (Carlsbad, USA). In brief, PANC-1 cells were cultured in Dulbecco’s modified Eagle’s medium (DMEM; Hyclone, Logan, USA) containing fetal bovine serum (FBS; Gibco, Carlsbad, USA) in a final concentration of 10%. SW1990 cells were cultured in Leibovitz medium (L-15; Hyclone) containing 10% FBS.

### Lentivirus production and stable cell line selection

To generate shRNA expression constructs against ARF6 and DUSP6, pLKO.1 TRC cloning vector (Addgene plamid: 10878; Addgene, Cambridge, USA) was employed [23]. Targets (21 bp) against ARF6 were as follows: sense, 5′-GCTCACATGGTTAACCTCTAA-3′ and antisense, 5′-AGCTGCACCGCATTATCAATG-3′. Targets (21 bp) against DUSP6 were as follows: sense, 5′-CTGTGGTGTCTTGGTACATTG-3′ and antisense, 5′-TCTAATCCAAAGGGTATATTT-3′. Overexpression constructs of DUSP6 were obtained by using the pCDH-CMV-MCS-EF1-Puro vector, and empty vector (EV) was used as the control. Lentiviral particles of ARF6 were produce by co-transfection of pLKO.1-shARF6 constructs with psPAX2 and pMD2.G into HEK-293T cells in a ratio of 4:3:1. It is the same when it comes to the DUSP6. Cell lines were obtained by infection of PANC-1 and SW1990 cells with lentiviral particles, followed by puromycin selection.

### Small interfering RNA

SiRNA duplexes against DUSP6 were transfected into pancreatic cancer cells using Lipofectamine 2000 (Invitrogen). The siRNAs were purchased from Shanghai Tuoran Biotechnology (Shanghai, China). The duplex sequences were as follows:si-DUSP6-1: 5′-GCAGCGACUGGAACGAGAAdTdT-3′ (forward), and 5′-UUCUCGUUCCAGUCGCUGCdTdT-3′ (reverse); si-DUSP6-2: 5′-GCAUUGCGAGACCAAUCUAdTdT-3′ (forward), and 5′-UAGAUUGGUCUCGCAAUGCdTdT-3′ (reverse). The sequence of control siRNA was as follows: 5′-UUCUCCGAACGUGUCACGUTT-3′ (forward), and 5′- ACGUGACACGUUCGGAGAATT-3′ (reverse).

### Cell viability assay

CCK-8 (Cell Counting Kit-8; Dojindo Laboratories, Tokyo, Japan) was used to detect cell viability according to the manufacturer’s instructions. In brief, cells were plated in 96-well plates at a density of 5×10 ^3^ cells per well and cultured at 37°C with 5% CO _2_ for 0, 24, 48, 72 and 96 h. Cells were then incubated with 10 μl cell counting kit-8 (CCK-8) reagent per well for 2 h. Absorbance at 450 nm was measured with a microplate reader.

### Colony formation assay

Cells in the logarithmic growth phase were seeded at 8×10 ^2^ cells per well in a 6-well plate. Colony formation was assessed by observing the cells under an inverted microscope (Olympus Corporation, Tokyo, Japan) for 2 weeks. Cells were washed, fixed with 4% paraformaldehyde for 10 min, and stained with 1 mL 0.1% crystal violet for 10 min. The remaining crystal violet was washed away with double distilled water. The numbers of colonies were counted after air-dried and photographed. All samples were assayed in triplicates.

### RNA isolation and sequencing

Total RNA was extracted from shNC and shARF6 cells using the Trizol (Invitrogen).For RNA-seq, shNC and shARF6 cells were utilized to analyze differentially expressed genes (DEGs). The adaptor sequences were removed from Illumina sequencing reads firstly. The raw Illumina sequence data were then demultiplexed and converted to FASTQ files, and adaptor and low-quality sequences were quantified. Reads were mapped to the hg38 human genome reference and underwent significant QC steps.

### Quantitative real-time PCR

Total RNA was extracted from cells using Trizol reagent (Invitrogen). cDNA was synthesized using the PrimeScript RT Reagent kit (TaKaRa, Dalian, China). Quantitative real-time PCR was conducted as described previously [24]. The relative gene expression levels were calculated using the 2 ^−ΔΔCt^ method, and normalized to that of *β-actin* which was used as an internal control. All the reactions were run in triplicate. All primer sequences are listed in Table 1.

**Table:** **Table 1** Sequences of primes used in this study

Gene	Forward sequence (3′→5′)	Reverse sequence (3′→5′)
*DUSP6*	GTGCCCTTCGCGTCGGAAAT	TGTCCCAAGGTCGTGTCGTCG
*CDX2*	CAGACTACCATCCGCACCACCAC	CTCAGGCCACAGAAGGGACGTTC
*DUSP8*	ATAAAGCCAAGCTCTCCAGCTGCC	CAGCAGCTGGCCCAGGAAGTTGAA
*SESN2*	AGTAGACAACCTGGCAGTGGTG	CTTCTGGTGGGCTTCTTACATG
*SIRT3*	GCGGCAGGGACGATGTGAG	ATCACCTCGAAGACCCGACCT
*RB1*	GTGCTGAAGGAAGCAACCCTCCT	GTCCACCAAGGTCCTGAGATCCTC
*CES1*	ATGTGGCTCCGTGCCTTTATCCTG	CGAAAGACCTGGTTGGAGAAACGG
*β-Actin*	CTACGTCGCCCTGGACTTCGAGC	GATGGAGCCGCCGATCCACACGG

### Western blot analysis

Cells were lysed on ice for 30 min in RIPA buffer (Beyotime, Shanghai, China), supplemented with protease inhibitors and protein phosphatase inhibitors (Roche, Basel, Switzerland). Total protein concentration was measured with BCA Protein Assay kit (Beyotime). Approximately 12 μg protein samples were separated on 8% or 10% SDS-polyacrylamide gels and then transferred to PVDF membranes (Millipore, Billerica, USA) for antibody incubation and color development. After being blocked with 5% fat-free milk for 1 h, membranes were incubated with primary antibodies overnight at 4°C. The antibodies used were as follows. Anti-β-actin antibodies were purchased from Proteintech (Rosemont, USA). Anti-ARF6 antibodies were purchased from Abcam (Cambridge, UK). Anti-DUSP6 antibodies were purchased from Abclonal (Beijing, China). Anti-44/42 MAPK (ERK1/2), phospho-p44/42 MAPK (ERK1/2) (Thr202/Tyr204), PARP, Cleaved PARP, Caspase 3 and BCL2 antibodies were purchased from Cell Signaling Technology (Beverly, USA). After extensive wash, the membranes were incubated with the corresponding horseradish peroxidase-conjugated secondary antibodies (Cell Signaling Technology) for 1 h at room temperature and detected using ECL solution (Thermo Fisher Scientific, Waltham, USA).

### Immunohistochemical staining (IHC)

Forty patients diagnosed with pancreatic cancer in Fudan University Shanghai Cancer Center (FUSCC) were randomly selected, and the clinical tissue samples were obtained with patients’ consents and approval from the Institutional Research Ethics Committee of FUSCC. Antibodies against ARF6 and DUSP6 were used to conduct immunohistochemical staining in paraffin-embedded tissues according to standard IHC procedures. Anti-ARF6 antibody (ab77581; Abcam) and anti-DUSP6 antibody (A3171; Abclonal) were used in a dilution of 1:1000. Three different views were randomly chosen under the microscope (Leica, Wetzlar, Germany) for scoring for each slide. Positive proportion and intensity were semiquantitatively scored according to the total area and intensity of the staining as previously described [25].

### Immunofluorescence microscopy

Cancer cells were seeded on culture slides at low density and cultured to 60%–70% confluency. For immune-staining, cells were washed twice with PBS, and fixed with 4% paraformaldehyde for 20–30 min at 4°C. Permeabilization was performed with 1% Triton-X100 for 5–15 min at room temperature after being washed twice with PBS. After wash with PBS, the slides were blocked with 1% BSA for  30 min. The antibodies used were anti-ARF6 and anti-DUSP6 (1:100 dilution) and were shown to be specific. Slides were incubated with primary antibody at 4°C overnight. After being washed with PBS, slides were incubated with Alexa-conjugated secondary antibodies (1:2000 dilution; Cell Signaling Technology) for 30 min at room temperature. Cells were then washed with PBS and the nuclei were stained with DAPI. Finally, the slides were observed under a fluorescence microscope (Leica).

### Statistical analysis

All statistical analyses were performed using GraphPad Prism 8 (San Diego, USA). *P*<0.05 was considered significantly different.

## Results

### Altered gene expression of DUSP6 in ARF6-knockdown cells is identified by RNA sequence and real-time PCR analysis

**Figure 1:**
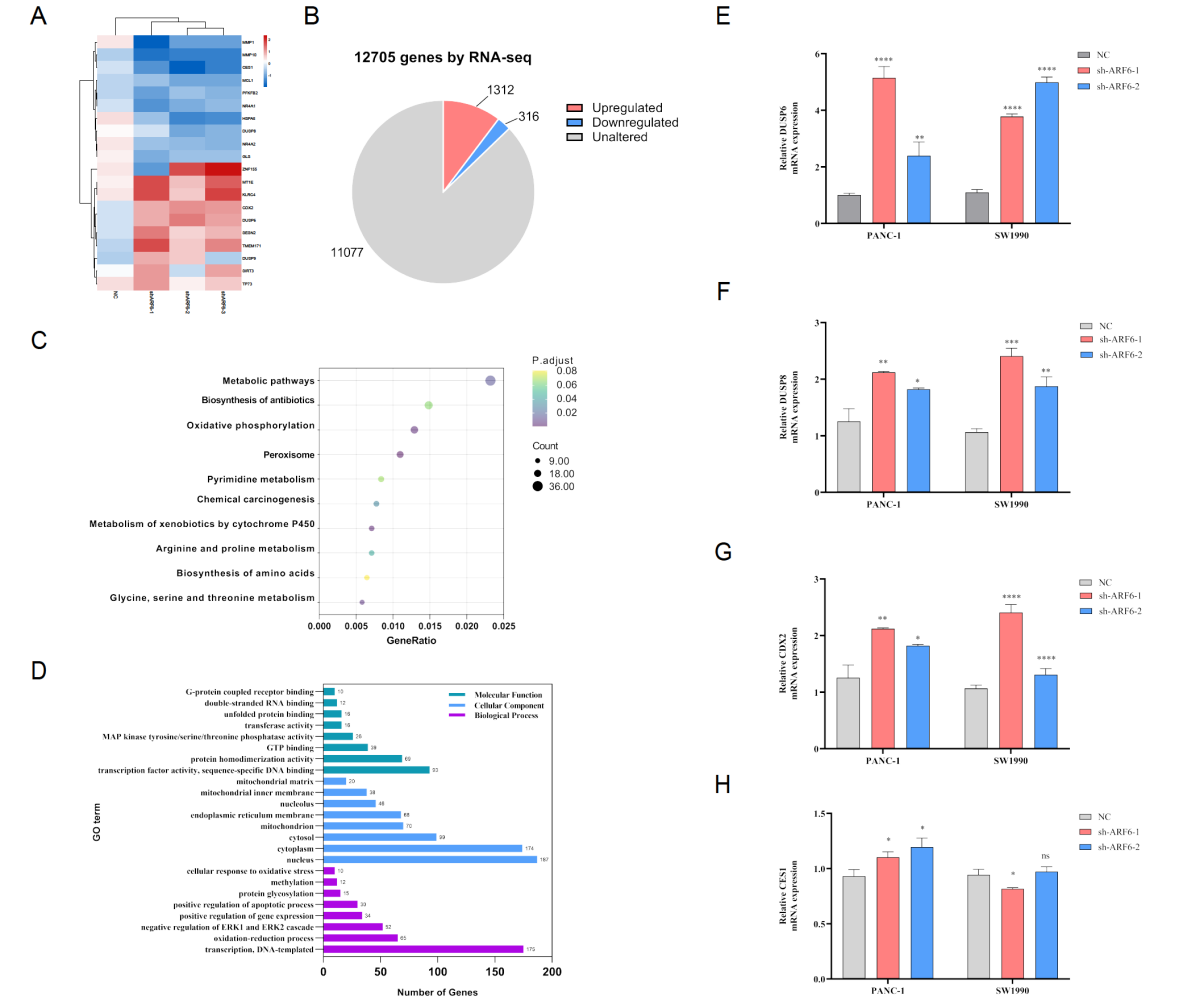
Figure 1 RNA sequencing and real-time PCR are used to identify the altered gene expression of DUSP6 in ARF6-knockdown cells (A) RNA sequencing was carried out to explore the differential gene expression and shown as heat map. (B) The number of upregulated and downregulated genes are shown. (C) KEGG analysis was performed to identify the significant signaling pathway. (D) Both the upregulated and downregulated genes were analyzed for GO term enrichment. (E−H) Validation of part of the RNA sequencing results by quantitative real-time PCR. * P<0.05, ** P<0.01, *** P<0.001, and **** P<0.0001.

To gain further insight into the molecular mechanisms responsible for the effects of ARF6, RNA sequencing was performed and the DEGs between negative control (NC) group and low ARF6 expression group were identified, among which 1312 genes were upregulated and 316 genes were downregulated ( Figure 1A,B). Kyoto Encyclopedia of Genes and Genomes (KEGG) analysis revealed that the cytokine–cytokine receptor interaction and MAPK signaling pathway were highly altered ( Figure 1C). Gene ontology (GO) enrichment analysis identified the top 8 DEGs covering three aspects of biology: cellular component, molecular function, and biological process ( Figure 1D). According to the analysis of DEGs, we found that the metabolic pathways are highly altered, which is consistent with our previous study [14]. In addition, in the “biological process” part, 52 genes are related to “negative regulation of ERK1 and ERK2 cascade”. This is well correlated with our primary concern in the present study. It has been confirmed that DUSP family has a close relationship with the MAPK pathway [19]. Thus, based on these results and the PCR results ( Figure 1E–H), *DUSP6* was considered as the candidate gene for further investigations.

### DUSP6 is negatively regulated by ARF6

**Figure 2:**
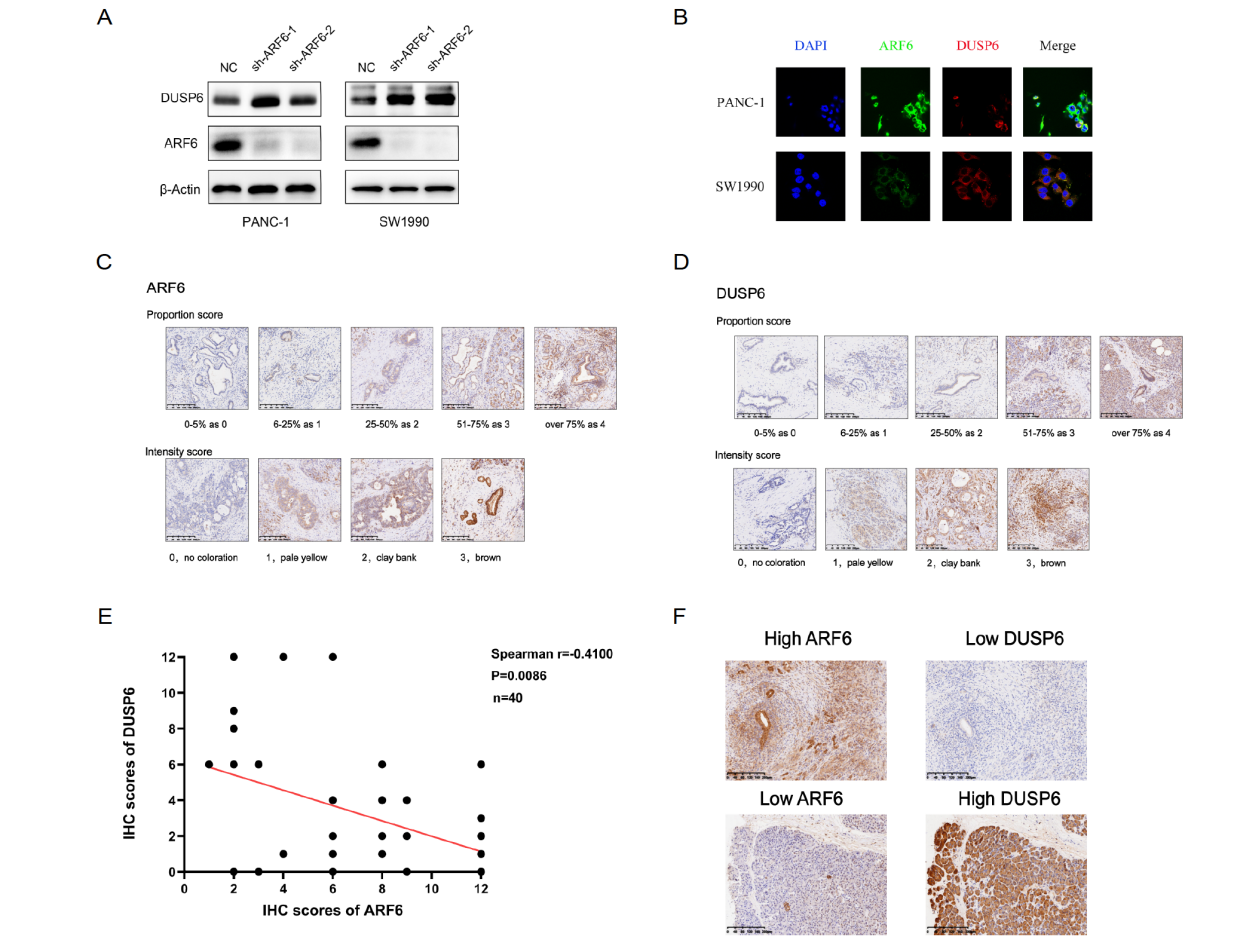
Figure 2 DUSP6 is negatively regulated by ARF6 (A) Knockdown of ARF6 in PANC-1 and SW1990 cells resulted in an increase of DUSP6 protein level. (B) Immunofluorescence microscopy analysis of DUSP6 and ARF6 expressions in PANC-1 and SW1990 pancreatic cancer cell lines. (C,D) Representative images showing proportion score and intensity score of ARF6 and DUSP6. (E) ARF6 is negatively correlated with DUSP6 expression in pancreatic cancer patients as demonstrated by immunohistochemical staining and scoring. (F) The correlation between ARF6 and DUSP6 expressions derived from clinical specimens. Patients with higher level of ARF6 displayed lower DUSP6 expression.

To further validate the relation between ARF6 and DUSPs, total protein was extracted and DUSP6 protein was analyzed by western blot analysis, Our resuts showed that DUSP6 had the same trend at protein level ( Figure 2A). To analyze the suitability of model cells for the follow-up experiments, we investigated two different human pancreatic cancer cell lines for their DUSP6 and ARF6 expressions ( Figure 2B). Based on the results, DUSP6 was found to be an important up-regulated target, which was selected for further experiments. For further verification of the observation between ARF6 and DUSP6 in patients’ tissues, IHC staining was conducted in 40 parafin-embedded tissue samples using antibodies against ARF6 and DUSP6. Moreover, the staining scores were calculated according to the method that multiplied the proportion and intensity scores. The scoring criteria of ARF6 and DUSP6 are listed in Figure 2C,D. After that, a statistical analysis of the correlation between ARF6 and DUSP6 was performed. A clear negative relation was found between ARF6 and DUSP6 in pancreatic cancer patients ( Figure 2E). Representative examples of opposite ARF6 and DUSP6 expressions are shown in Figure 2F. These results indicate that DUSP6 is negatively regulated by ARF6, and ARF6 is inversely correlated with DUSP6 in PDAC tissues, which has never been reported.

### DUSP6 silencing promotes cell growth and cell proliferation in human pancreatic cancer cells

**Figure 3:**
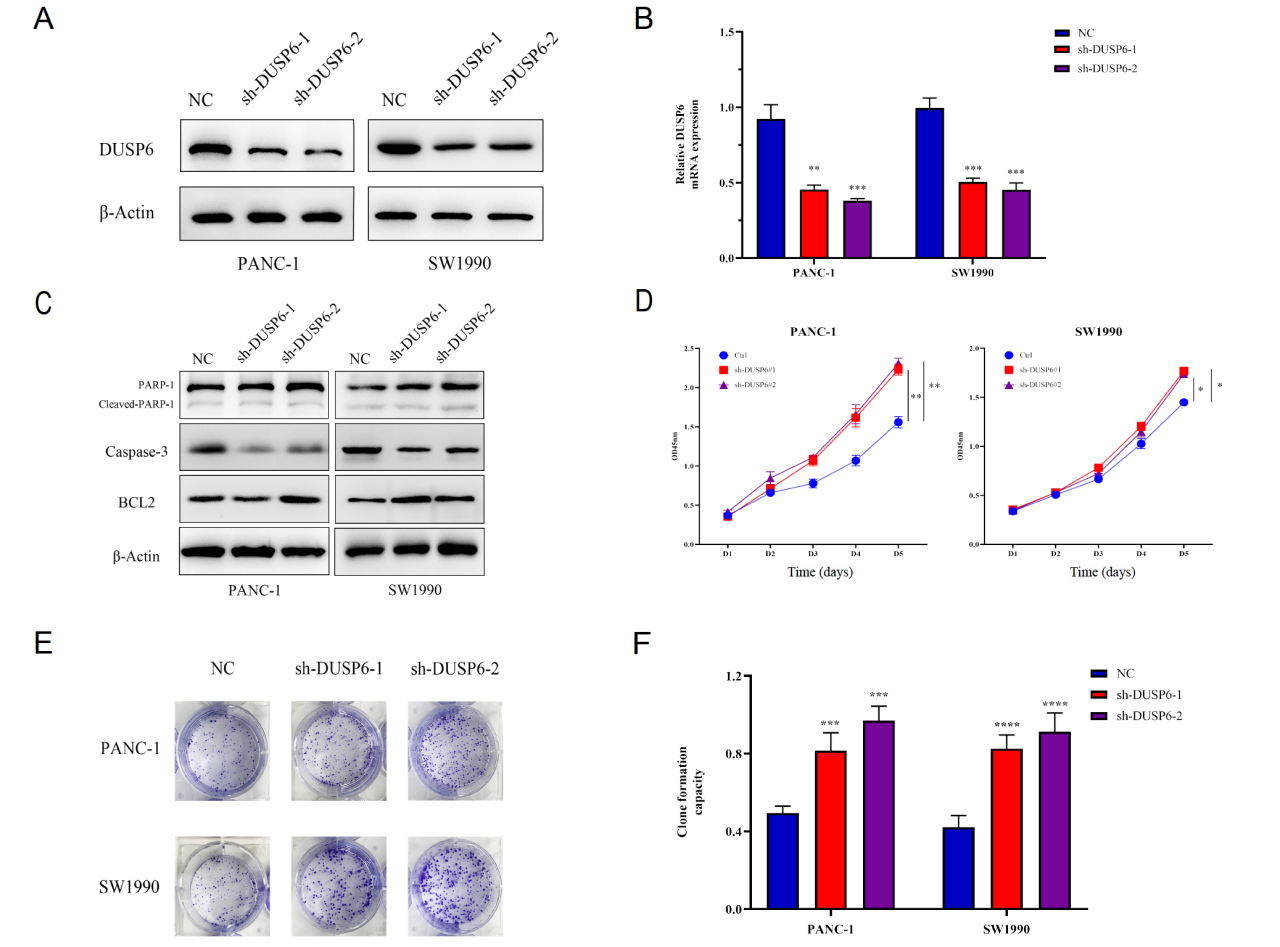
Figure 3 DUSP6 silencing promotes cell growth and cell proliferation in human pancreatic cancer cells (A,B) The shRNAs against DUSP6 plasmid were transfected into PANC-1 and SW1990 cells to examine the decrease of DUSP6 mRNA and protein levels, respectively. (C) Western blot analysis of apoptotic proteins Caspase-3, BCL2, PARP and cleaved PARP. (D) Silencing DUSP6 expression, as indicated in (A,B), decreased the proliferation of PANC-1 and SW1990 cells, as determined by CCK-8 assay. (E,F) Decrease of DUSP6 expression increased the colony formation ability of PANC-1 and SW1990 cells. * P<0.05, ** P<0.01, *** P<0.001, and **** P<0.0001.

We found that DUSP6 is negatively regulated by ARF6, which acts as an oncogene in PDAC. Meanwhile, the physiological role of DUSP6 has seldom been discussed in pancreatic cancer. Therefore, we further validated the function of DUSP6 in tumor proliferation. We firstly generated PANC-1 and SW1990 cell lines that stably express shRNAs against DUSP6. After that, the silencing effects were validated by real-time PCR and western blot analysis ( Figure 3A,B). To explore the role of DUSP6 in pancreatic cancer cell apoptosis, we first detected the apoptosis-related proteins, poly ADP-ribose polymerase (PARP) and cleaved PARP by western blot analysis. The results showed that there was no significant difference in the expressions of apoptotic proteins between the DUSP6 silencing group and the negative control (NC) group ( Figure 3C). Next, a series of function experiments were performed using the knockdown cell lines to elucidate the DUSP6 gene function. CCK-8 assays showed that the proliferation of both DUSP6-knockdown cell lines was increased, and the increase of PANC-1 cells was more significant ( Figure 3D). Furthermore, the colony formation assay results showed that the ratio of colony formation of both DUSP6-knockdown cell lines was much higher than that of the NC group, which indicated that DUSP6 silencing promoted colony formation capacity of SW1990 and PANC-1 cells ( Figure 3E,F). The proliferation assays *in vitro* further validated the role of DUSP6 in the proliferation of PDAC.

### Upregulated expression of DUSP6 reduces cell growth and induces apoptosis of pancreatic cancer cells

**Figure 4:**
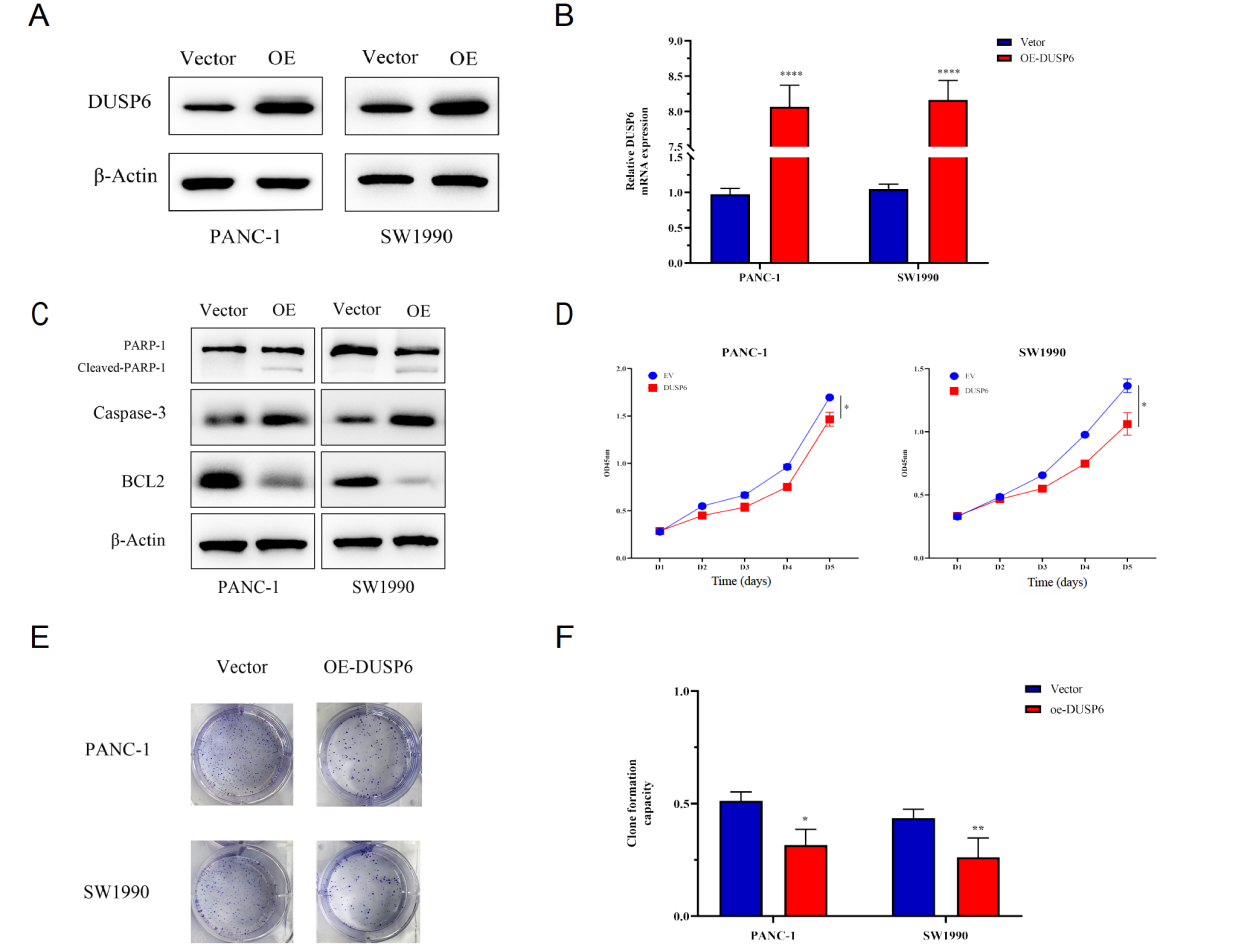
Figure 4 Upregulated expression of DUSP6 reduces cell growth and induces apoptosis of pancreatic cancer cells (A,B) qPCR and western blot analysis confirmed the overexpression (OE) efficiency of DUSP6 in PANC-1 and SW1990 cells. (C) Western blot analysis of apoptotic proteins PARP, Caspase-3 and BCL2. (D) Overexpression of DUSP6, as indicated in (A,B), increased the proliferation of PANC-1 and SW1990 cells, as determined by CCK-8 assay. (E,F) Overexpression of DUSP6 weakened the colony formation ability of PANC-1 and SW1990 cells. * P<0.05, ** P<0.01, and **** P<0.0001.

Similarly, to further validate our findings, the same cell lines overexpressing DUSP6 were constructed, which showed favorable overexpression efficiency ( Figure 4A,B). Because low DUSP6 expression occurred more frequently in highly invasive and poorer differentiated cancers [26], we detected the apoptosis-related proteins in the cell lines overexpressing DUSP6. Surprisingly, the effect of DUSP6 overexpression on cleaved PARP protein level was significantly increased ( Figure 4C). To investigate the impact of DUSP6 overexpression on the proliferative capability of pancreatic cancer cells, cell proliferative capability was examined by CCK8 assays and the colony formation assay. The results of CCK8 assay showed that the proliferative capability was severely impaired in the DUSP6 overexpression (oe-DUSP6) group compared with that in the Vector group at day 3, day 4 and day 5 ( Figure 4D). The colony formation assay results showed that the colony formation rate of the oe-DUSP6 group was 51.27%±5.81% (PANC-1) and 43.64%±5.45% (SW1990),which was higher than those of their corresponding Vector group (26.18%±8.00% in PANC-1 and 31.64%±8.00% in SW1990) ( Figure 4E,F). These results showed that DUSP6 reduces pancreatic cancer cell growth, and promotes the apoptosis of cancer cells *in vitro*.

### ARF6 regulates ERK1/2 activation and pancreatic cancer cell proliferation via DUSP6

**Figure 5:**
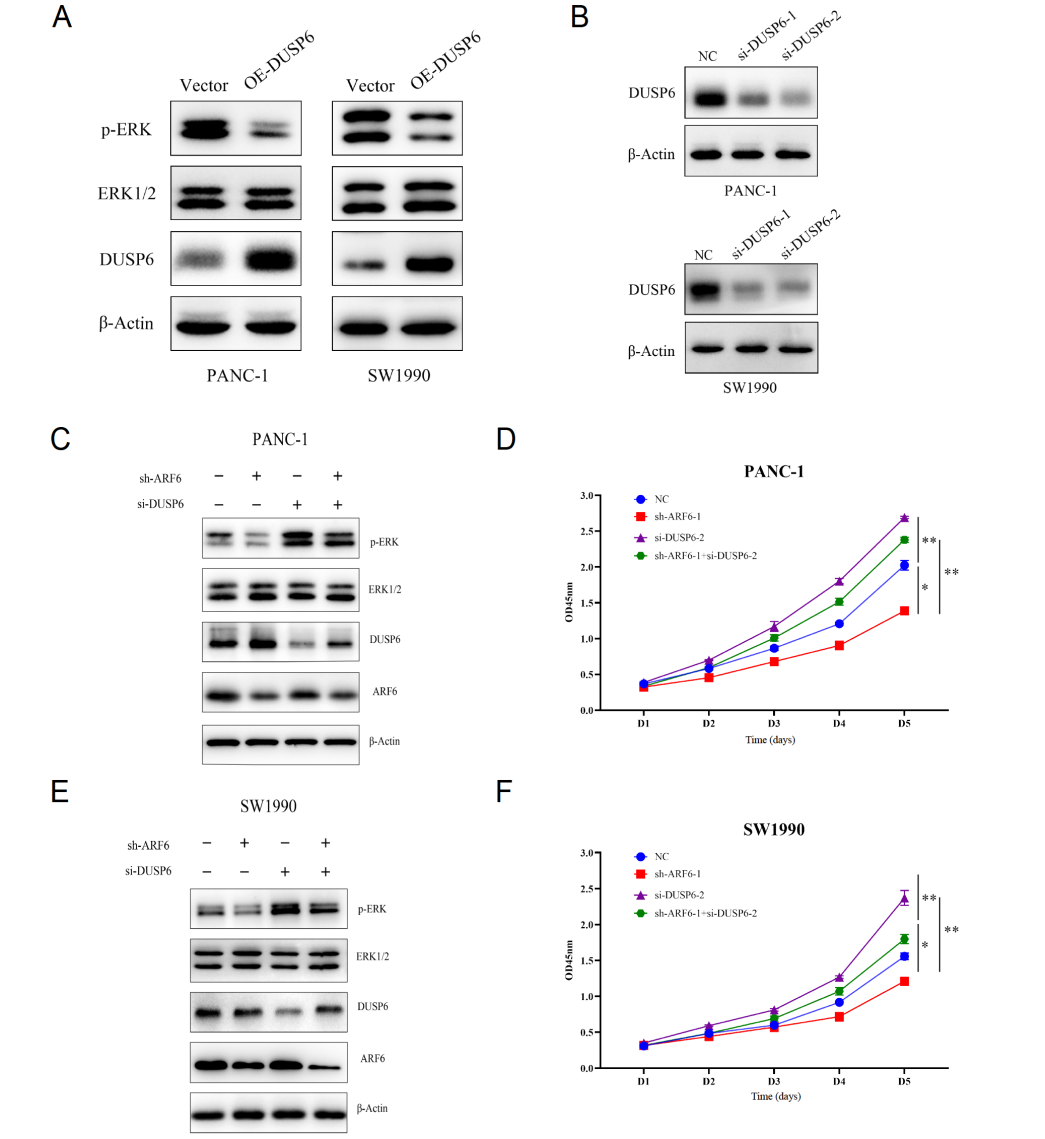
Figure 5 ARF6 regulates ERK activation and pancreatic cancer cell proliferation via DUSP6 (A) Overexpression (OE) of DUSP6 suppressed ERK activation at protein levels. (B) The siRNA against DUSP6 was transfected into the PANC-1 and SW1990 cells to examine the decrease of DUSP6 protein level. (C,E) ERK and p-ERK expression levels were examined by western blot analysis in ARF6- and/or DUSP6-knockdown cells. (D,F) Knockdown of ARF6 and/or DUSP6 manifested different proliferation of PANC-1 and SW1990 cells, as determined by CCK-8 assay. * P<0.05, and ** P<0.01.

Since ARF6 directly targets DUSP6, and our results showed that ARF6 enhances the activation of ERK1/2, we then tested whether DUSP6 activates the ERK1/2 pathway. We found that ERK1/2 activation was inhibited at the protein level after overexpressing DUSP6 ( Figure 5A). We next explored whether ARF6 affects the activation of ERK1/2 via inhibiting DUSP6. siRNAs were used to inhibit DUSP6 in ARF6-knockdown cells and the activation status of ERK1/2 pathway was tested. The interference efficiencies of two sequences of si-RNA targeting DUSP6 in PANC-1 and SW1990 cells were evaluated by western blot analysis ( Figure 5B), and si-DUSP6-2 which exhibited higher efficiency was selected for the subsequent experiments. The results demonstrated that knockdown of DUSP6 could reverse the inhibition of the ERK1/2 pathway caused by ARF6 knockdown ( Figure 5C,E). Moreover, CCK-8 assay results indicated that knockdown of DUSP6 promoted the proliferation of the ARF6-knockdown cells ( Figure 5D,F). This phenomenon was particularly evident in the PANC-1 cell line, in which the proliferation rate of the double knockdown cells was found to proliferate slightly faster than that of the NC cells ( Figure 5D). These results support our hypothesis that ARF6 regulates ERK1/2 activation and pancreatic cancer cell proliferation via DUSP6.

**Figure 6:**
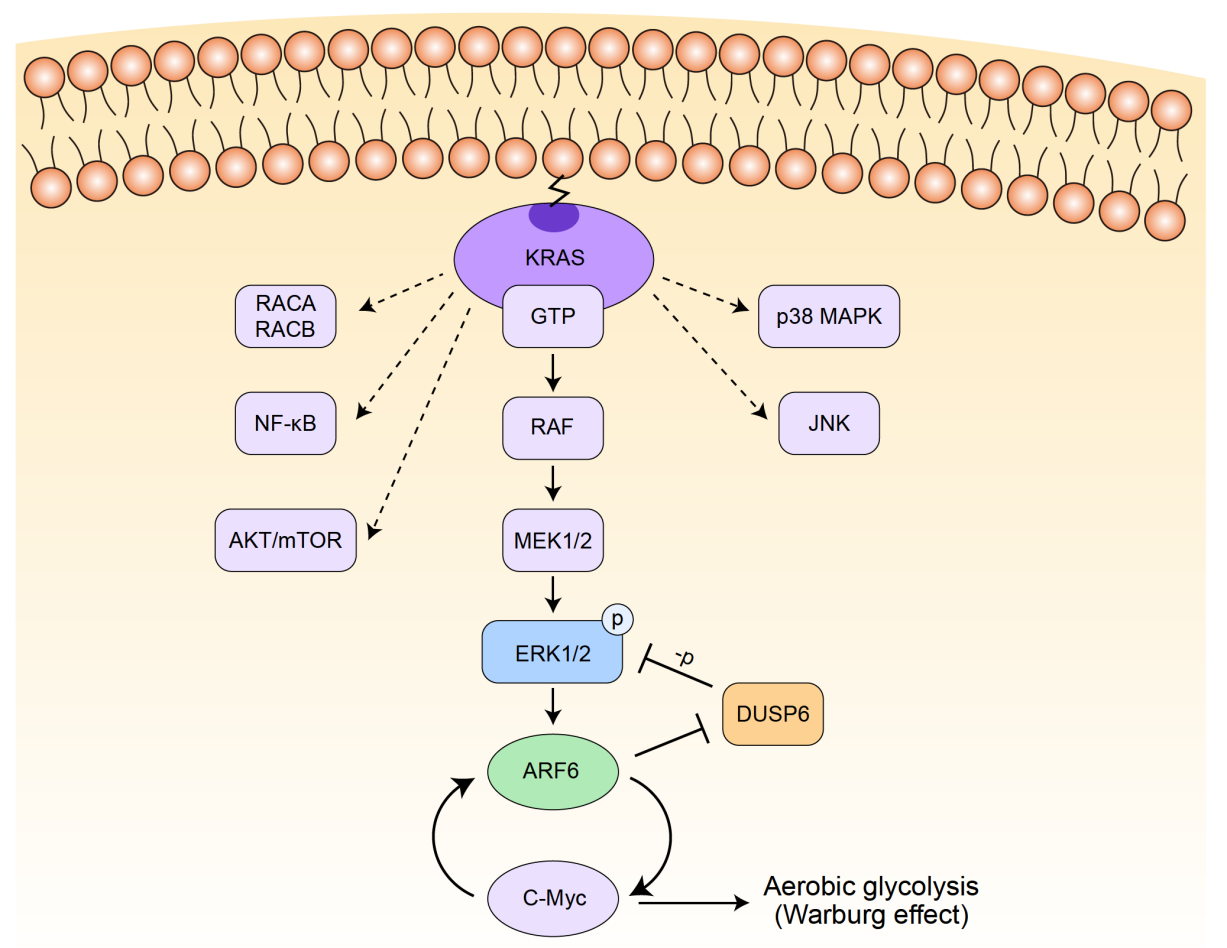
Figure 6 Schematic representation of the model of the positive feedback loop of ARF6 KRAS activates ARF6 via ERK1/2, which in turn leads to the inhibition of DUSP6, which causes the attenuation of ERK1/2 inhibition in the positive feedback loop. DUSP6 acts both as a tumor suppressor in pancreatic cancer and as a vital intermediate molecule in the positive feedback loop.

In conclusion of the whole study, we demonstrated that a pathway driven by KRAS, activates ARF6 via ERK1/2, which in turn leads to the inhibition of DUSP6, which causes the attenuation of ERK1/2 inhibition in our positive feedback loop. DUSP6 can not only act as a tumor suppressor in pancreatic cancer, but also as a vital intermediate molecule in our positive feedback loop composed of ERK1/2, ARF6 and DUSP6 ( Figure 6). However, further studies are required to determine whether multiple pathways are involved in the positive feedback effect.

## Discussion

Pancreatic cancer is an aggressive malignant digestive tract tumor. Because of the small chance of accepting an operation and the existence of chemoresistance, targeted therapy or immune therapy is needed to treat pancreatic cancer. Our experiment is based on *KRAS* which is the most common mutated gene in pancreatic cancer cells. Even though the *KRAS* gene was discovered decades ago, no effective therapy for KRAS or its downstream molecules has been applied to clinical use [27]. Therefore, to improve the poor overall survival rate of pancreatic cancer patients, it is urgent to explore the downstream effector molecules of KRAS. Based on previous studies, we speculate that there is a positive feedback loop that is probably involved in ARF6 and ERK1/2 pathways which are the downstream signaling molecules of KRAS [14]. The present study was designed to determine the existence of yet new positive feedback loop and the related molecular mechanisms. We believe that the presence of this positive feedback loop may be the reason for the uncontrolled proliferation and the malignancy properties maintenance of pancreatic cancer cells. Here, we screened and detected the downstream signaling molecules of ARF6 using RNA sequencing and q-PCR. We focused on the DUSPs family, in particular DUSP6 and DUSP8, with significantly differential expressions. Previous studies have demonstrated that the dephosphorylation of the DUSP family inhibited the ERK1/2 pathway in pancreatic cancer [ 17, 26] . However, no relevant result has been reported on the regulation of DUSPs by ARF6 and their contribution to the KRAS-related positive feedback loop. Furthermore, our analysis of PDAC tumor specimens demonstrated that high ARF6 level is correlated with low DUSP6 level.

Previous reports suggested that ARF6 plays essential roles in various essential biologic processes, especially in regulating the Warburg effect that provides continuous energy and nutrients for uncontrolled proliferation in tumors [14]. As a result, we observed significant changes in the proliferation of PDAC cells after knockdown or overexpression of DUSP6. Our results suggest that ARF6 may promote the growth of pancreatic cancer cells through DUSP6. Considering the extensive proliferation of pancreatic cancer, elaborating the relationship between ARF6 and DUSP6 in pancreatic cancer may bring a new strategy for treating this recalcitrant tumor.

DUSP6 is a member of the protein-tyrosine phosphatase family, which inactivates their target kinases by dephosphorylating both the phosphoserine/threonine and phosphotyrosine residues and negatively regulate members of the MAPK superfamily that is linked to cell proliferation and differentiation [28]. As shown in our results, DUSP6 is negatively regulated by ARF6, thus weakening its dephosphorylating and inhibiting effect on the ERK1/2 pathway. Through immunohistochemistry, we also found a negative correlation between the expressions of ARF6 and DUSP6 in tissues. In addition, we investigated the biological functions of DUSP6 by gain-of-function and loss-of-function experiments, since these results are consistent with recent studies demonstrating that DUSP6 acts as a tumor-suppressor gene in pancreatic cancer [26]. Meanwhile, the levels of cleaved-PARP and Caspase-3 proteins in pancreatic cancer cells with overexpression of DUSP6 were significantly increased, while the BCL2 protein was decreased, suggesting that DUSP6 promotes the apoptosis of pancreatic cells. Surprisingly, there was no significant difference in the expression of PARP protein between the DUSP6 silencing group and control group. However, Caspase-3 protein was decreased and BCL2 was slightly increased following DUSP6 knockdown. These findings suggest that there is no significant change in the apoptotic ability in DUSP6 knockdown cells. In order to elucidate that ARF6 regulates ERK1/2 pathway activation via DUSP6, we down-regulated both *ARF6* and *DUSP6*, and compared with untreated cells or cells with single gene low expression, and finally came to the conclusion.

Indeed, despite over 95% of pancreatic cancers harbor oncogenic *KRAS* mutations, and the ERK1/2 signaling pathway is always involved in this process, other gene mutations, such as *TP53* and *SMAD4* which often exist in PDAC patients, may also have a promoting or inhibiting effect on tumors by regulating the activation of the ERK1/2 signaling pathway [ 29, 30] . In addition, *KRAS* mutation has been found in approximately 30% of all human cancers and is assumed to play a critical role in tumor occurrence and progression [31], which further explains the applicability and generalizability of the positive-feedback loop.

The limitation of this study is that we focus narrowly on the role of DUSP6 without considering the part of other downstream ARF6 molecules. It has been confirmed that ARF6 is localized to the plasma membrane (PM) and vesicle membrane in the cytoplasm [32]. Meanwhile, DUSP6 is mainly concentrated in the nucleus. Thus, further studies are required to explore what the transcription factors that mediate ARF6 to inhibit DUSP6 expression are. The effect of DUSP6 expression on prognosis and chemotherapy tolerance of pancreatic cancer patients also needs to be further investigated.

In conclusion, this study reveals the role of DUSP6 in the ARF6-ERK1/2 positive feedback pathway, and verifies the effects of DUSP6 on the growth, proliferation and apoptosis of pancreatic cancer cells. We demonstrated that a pathway driven by KRAS activates ARF6 via ERK1/2, which in turn leads to the inhibition of DUSP6, which causes the attenuation of ERK1/2 inhibition in our positive feedback loop. DUSP6 can act not only as a tumor suppressor in pancreatic cancer, but also as a vital intermediate molecule in our positive feedback loop composed of ERK1/2, ARF6 and DUSP6 ( Figure 6). Our results provide potential predictive and therapeutic targets for pancreatic cancer. However, further studies are required to determine whether multiple pathways are involved in the positive feedback effect.

## Supporting information

21670Table_1
