# Supplementary material for: A positive feedback loop of ARF6 activates ERK1/2 signaling pathway via DUSP6 silencing to promote pancreatic cancer progression : ARF6 regulates ERK1/2 pathway by inhibiting DUSP6
Source: Acta Biochim Biophys Sin (Shanghai). 2022 Aug 19;54(10):1431–40. doi: 10.3724/abbs.2022111 (PMC9827993; doi:10.3724/abbs.2022111)
Supplement: 21670Table_1 [file 21670Table_1.pdf]

Table 1. Sequences of primes used in this study

Gene	Forward (3′→5′)	Reverse (3′→5′)
DUSP6	GTGCCCTTCGCGTCGGAAAT	TGTCCCAAGGTCGTGTCGTCG
CDX2	CAGACTACCATCCGCACCACCAC	CTCAGGCCACAGAAGGGACGTTC
DUSP8	ATAAAGCCAAGCTCTCCAGCTGCC	CAGCAGCTGGCCCAGGAAGTTGAA
SESN2	AGTAGACAACCTGGCAGTGGTG	CTTCTGGTGGGCTTCTTACATG
SIRT3	GCGGCAGGGACGATGTGAG	ATCACCTCGAAGACCCGACCT
RB1	GTGCTGAAGGAAGCAACCCTCCT	GTCCACCAAGGTCCTGAGATCCTC
CES1	ATGTGGCTCCGTGCCTTTATCCTG	CGAAAGACCTGGTTGGAGAAACGG
β-Actin	CTACGTCGCCCTGGACTTCGAGC	GATGGAGCCGCCGATCCACACGG
